# Trends in Social Acceptance of Renewable Energy Across Europe—A Literature Review

**DOI:** 10.3390/ijerph17249161

**Published:** 2020-12-08

**Authors:** Marco Segreto, Lucas Principe, Alexandra Desormeaux, Marco Torre, Laura Tomassetti, Patrizio Tratzi, Valerio Paolini, Francesco Petracchini

**Affiliations:** 1 National Research Council of Italy, Institute of Atmospheric Pollution Research, via Salaria 29300, 00015 Monterotondo, Italy; m.segreto@iia.cnr.it (M.S.); m.torre@iia.cnr.it (M.T.); l.tomassetti@iia.cnr.it (L.T.); p.tratzi@iia.cnr.it (P.T.); petracchini@iia.cnr.it (F.P.); 2 College of Engineering, Northeastern University, 360 Huntington Ave, Boston, MA 02115, USA; principe.l@husky.neu.edu; 3 Department of Geography and Environmental Studies, Faculty of Art and Social Sciences, Carleton University, 1125 Colonel By Drive, Ottawa, ON K1S 5B6, Canada; AlexDesormeaux@cmail.carleton.ca

**Keywords:** social acceptance, public involvement, renewable energies, climate change, biogas, wind energy

## Abstract

Social acceptance has proven to be a significant barrier in the implementation of renewable energy systems (hereinafter “RES”). While a general acceptance of RES is high, low local acceptance has hindered the development of renewable energy projects (hereinafter “REP”). This study assesses the determinants of local and general social acceptance of REP across Europe through a qualitative analysis from 25 case studies of the most significant social drivers and barriers that include all European countries. These case studies contain qualitative and quantitative analyses of the main factors for social acceptance of many representative groups including residents, stakeholders, and experts. Understanding the influences of social acceptance enables us to create strategies that will promote the development of REP by mitigating any public opposition.

## 1. Introduction

Due to the threat of climate change, the growing concerns on upward trends in emissions of climate-forcing atmospheric pollutants (i.e., CO_2_ and CH_4_) and the need to secure energy independence has led the European Union to negotiate a new Renewable Energy Directive (hereinafter “RED II”) in 2018 to solicit its Member States to undertake a concerted action aiming to transform Europe into a global leader in a variety of renewable energy sectors. To curb the threat of anthropogenic climate change, the European Union (hereinafter “EU”) has approved policies aiming to encourage private investments in expanding the renewable energy production capacity in Europe. The RED II directive modified its previous goal of 20% total energy generation from renewables across the EU by 2020, to 27% by 2030 [1].

Furthermore, the climate and energy framework require by 2030 a minimum 40% decrease in greenhouse gas emission levels and an improvement in energy efficiency by 27%.

While these targets may seem too ambitious at first, it is important to realize that the RED II calls only for total energy production of 27% across the entire European Union by 2030; this does not mean that each Member State needs to supply 27% of their energy from renewables by 2030. Therefore, different countries, with different energy infrastructures and histories, have pledged different targets by that time. Nonetheless, each country in the EU has been pushed to significantly develop more renewable energy (RE) infrastructures. With this political will through a strong governmental pressure in place, other challenges sometimes arise in the completion of a renewable energy projects (REP). Social acceptance is one of the most important limiting factors that regularly delay the installation and operation of renewable energy plants.

In 2018, the European Parliament and European Council have agreed again on a further binding target for 32% of energy use that shall come from renewable resources in the European Union by 2030, following more than a year and a half of negotiation. Although the 32% target is binding on the EU as a whole, there are no national targets, and enforcement will depend on the Energy Union Regulation which is currently being negotiated.

While the global benefits of renewable energy is well known, some concerns still exist on their impact on local environment [2,3]. Despite non-renewable energy sources generally have an even worse environmental impact [4,5], the overall uncertainty related to the local impacts of renewable energy plants negatively affect the social acceptance. Social acceptance, defined as the active or passive approval by the public of a certain policy (Bertsch et al., 2016), is one of the most significant barriers toward achieving renewable energy targets. A distinction may be made between a “general social acceptance”, which is social acceptance on the broadest level and that may also be called socio-political acceptance, and a “local social acceptance”, which is active at a community level and is involved in siting and in the actuation of renewable energy projects [6].

In general, in many European countries, the rate of public acceptance across renewable energy sources has been measured to be significantly high [6,7,8,9,10]. In countries with high levels of general public acceptance, across many energy technologies, it has been observed that when one’s local community is directly impacted by the construction of a renewable energy plant, a lack of local community acceptance may grow and contribute to the failure of many promising renewable energy projects, some of which have been the subject of specific case studies [11,12,13]. In other instances, a variety of relational factors that contribute to forming social acceptance, including the trust in public authorities, distribution of quality information, public involvement, and economic benefits are important steps in the acceptance of REP across Europe [6,11,12,13,14,15,16].

While several European cities started a process of urban regeneration in the post-Fordist period following the pivotal examples of Barcelona [17] and Bilbao [18], the apparent improvements in better and healthier environments did not translate into solutions for renewable energy production. Behind these processes, instead, the creation of profit-driven spaces was the main focus of the interventions and social acceptance was usually limited to the upper class in order to create a proper social environment, thus leading to gentrification, people displacement and even more pollution following the logic of capital [19]. Even more recently, after the pandemic outbreak, these issues are under a specific treatment through new proposals that refer to the concept of 20-min neighborhoods, 15-min city, and superblocks, which generally seem to be more socially accepted than more effective solutions related to renewable energy projects [20,21].

This study aims to identify the trends in local and general social acceptance of renewable energy projects in Europe in order to create a better understanding of the general trends in local acceptance in REP across Europe and develop a framework that will reduce the probability that REP will face public opposition thereby facilitating the implementation of renewable energy systems (RES) by local and regional governments, and by private developers. This study will make a generalization on the public’s willingness to accept REP in their local communities.

This study includes the qualitative analysis from 25 case studies to identify the prominent drivers of local and general social acceptance of renewable energy projects in communities in Europe. The structure of this paper is as follows: the methodology, the case studies, the discussion, and the conclusion. We will first define and justify the methodology for this research paper. In the second part of the paper, we will discuss the prominent drivers of social acceptance evidenced by the twenty-five studies in the following order: trust in governance and procedural justice, distributional justice, concerns related to siting, and the effect socio-demographic factors on social acceptance, which are identified in [6] as the main areas involved in defining social acceptance at a general and local level. Following is a discussion of the results that will expand on the previous ideology and contextualize the drivers of social acceptance. We will also discuss the policy implications of the results. The last section of this paper will discuss the various strategies that can improve local acceptance of RES and minimize the risk of rejection. Most of the literature on social acceptance of RE focuses on the acceptance of a community in one or few European States. This study compiles evidence from multiple case studies to make a generalization of the determinants of local acceptance of renewable energy projects.

## 2. Materials and Methods

The research methodology used in this study for a theoretical generalization of the trends in social acceptance of renewable energy in Europe is the comparative method. Comparative case studies are useful for matters of human–environment interactions (Knight, 2001). In this study, we are assessing the drivers of a specific human behaviour (i.e., acceptance) to the implementation of renewable energy. This method allows the study of this specific human behaviour across the continent throughout the last two decades. A comparative method is a classical mode of analysis in anthropology, human geography, and sociology (Knight, 2001). The study intersects sociology with environmental science. A comparative case study brings an in-depth understanding of the drivers of social acceptance in different circumstances. This study uses secondary data that has already been collected (i.e., case studies) and performs a qualitative analysis which is often used in comparative case studies (Knight, 2001). Secondary data is used over primary data because it spans a longer timescale, in our specific case going from 2001 to 2019, and a broader geographical region and can more efficiently answer the research question: “What are the trends of the local social acceptance of renewable energy in Europe?”

This comparative case study can be considered a quality comparative analysis of existing literature: 25 studies relating to the public acceptance of renewable energy sources in Europe. This comparative case study is developed on the basis of geographical transects and includes secondary data from different spatial scales that provide information that can be generalized across a region, specifically Europe. Much of the literature suggests that the number of case studies required depends on the criteria for the studies and the nature of the data. There is no correct number of case studies defined for comparative research. However, a theoretical generalization requires a sufficient number of cases to evaluate the drivers of social acceptance under various conditions. Another comparative case study on the social acceptance of renewable energy included only five case studies in their analysis (Schumacher et al., 2019). The authors conducted a quantitative analysis and compared the results to other case studies to create a generalization of the social acceptance trends in that region. From the existing publications on social acceptance of renewable energy, the case studies selected are all within twenty years of publication, relate to the public’s acceptance of the implementation of a renewable energy project or specific renewable energy technology, and assess the behaviour of a European population. Following this selection process, 25 case studies were chosen for this comparative research from the available publications related to the topic. At the time of selection these 25 scientific papers were the whole of what the authors could find on the topic of social acceptance of renewable energy sources. A search on scientific literature search engines, such as SCOPUS, Google Scholar, could not find papers when the search string was “social acceptance” plus “renewable energy”. The search was also widened to include every combination of “renewable energy, solar energy, photovoltaic, wind energy, biomass, biogas” with either “acceptance” or “Not in My Backyard Phenomenon” (NIMBY).

There could very well be more case studies outside the search parameters described; however, this grouping of case studies is considered sufficient to make a sound theoretical generalization on the social acceptance trends of renewable energy in Europe. Listed in Table 1 are the case studies used in this analysis, together with the European population or community that was assessed which includes at least one case study that analysed each an eastern, western, central, and southern European population.

The comparative markers used to assess the selected case studies were chosen following the main parameters in [6] as they can be considered the defining features of general and local acceptance.

## 3. Results

The following section will cover the main determinants in the social acceptance of renewable energy technology.

### 3.1. Trust in Governance and Procedural Justice

Previous studies have shown that trust is a crucial aspect in determining the level of social acceptance. Per Table 2, more recent studies confirm the correlation between procedural justice, trust, and a greater social acceptance rate of REP.

A transparent process and the dissemination of information improve the level of mutual trust between the developer and the community, thereby increasing social acceptance. Distrust in the community has been shown to often be caused by a lack of knowledge on the efficacy of renewable energy and the process of development. Distrust also creates a negative perception of REP for the community. The studies indicate that community participation helps alleviate the community’s concerns and increase the level of mutual trust. Disseminating information is crucial to correct any misconceptions about RES and REP. It is also important to mitigate the concerns about siting, environmental and human ecological imprint, and the perceived benefits and costs.

### 3.2. Distributional Justice

It was found that perceived benefits and costs should be distributed equally amongst residents. Per Table 3, the studies reveal that a great incentive for local acceptance is a financial benefit for the inconvenience of developing a RES or REP in the community. For instance, lower energy rates, opportunities for employment, or tax returns. The research does not corroborate the claim from a study conducted in earlier years that residents would pay higher taxes to obtain green energy except for one study which suggests that residents would pay more for solar-generated energy [10].

However, much of the evidence suggests the opposite in that residents expect financial benefits or a significant community benefit. While community support by developers positively influences local acceptance, a financial incentive is shown to be the greatest motivator in accepting REP. It is also suggested that the perception of benefits and costs can be crucial. For instance, creating employment for the community is uncommon for REP developers however, offering a few jobs can affect the perception of the community to view the REP as an opportunity for financial gain and other benefits.

### 3.3. Siting

Per Table 4, many issues related to siting are unique to REP and are caused by its specific physical characteristics. For instance, a common concern regarding biogas is the disrupting odour. Another frequent complaint regarding the physical characteristics of wind farms is the noise pollution. These findings suggest that further effort is needed to disseminate the knowledge in the best practices to alleviate these concerns. In relation to the disrupting odour of biogas REP, several successful applications of odour control technologies are reported in recent literature, showing that smell can be efficiently avoided. Smells mostly depend on leakage and are affected by distance and wind speed [40]. Hydrogen sulfide (H_2_S) is generally the major contributor to unpleasant odours, due to its high concentration in biogas and relatively low olfactory threshold: this compound can also be efficiently removed by dosing air in the anaerobic digester. As for leakages, it has been recently reported that there are several preventative measures that can be taken to avoid them: a larger storage capacity, process optimization, and regular servicing. Concerning odor plume dispersion into the atmosphere, it is possible to easily prevent complaints through the application of odor predictions to the spatial planning of biogas plants [41].

Siting is one of the main determinants of social acceptance because it relates to many other concerns such as effects on human health and the environment. The research shows that generally, residents would not want to live near a renewable energy plant because it disturbs the aesthetic quality of the landscape. An additional common worry pertaining to smell is that it will decrease property values in the areas surrounding a biogas plant [12,14]. However, other literature suggests that there is no significant impact of the opening of a biogas plant on housing values in neighbouring areas [42]. This concern seems to be scientifically unjustified. Nevertheless, it still has been shown to weigh heavily on the decisions of residents.

### 3.4. Socio-Demographics

In addition to various specific reasons for public acceptance or rejection of renewable energy technologies, general demographics were also found to play a role in public perceptions according to Table 5. Preliminary demographic studies could help overcome public opposition and better formulate a strategy for an increased rate of acceptance of REP by knowing the size and composition of the target audience most likely to be biased or opposed. Based on this literature review, the trend indicates that higher levels of acceptance and awareness correlate positively with a higher level of education and a younger age.

Recent studies have not shown socio-demographic factors to be amongst the most institutional factors of social acceptance. The socio-demographic factors tend to have a small or medium effect on the local acceptance. The evidence corroborates earlier claims that older residents and residents with less education are less likely to accept REP. However, these concerns can easily be mitigated through participation models that include discussions with the community, panels, or other where locals can express their opinions.

## 4. Discussion

Results are discussed following the same categories employed in the rest of the paper, that is the prominent drivers identified in the literature [6]. For ease of reading, a brief summary of the discussion is provided in Table 6, highlighting the main positive actions that may be undertaken to increase social acceptance and the most common obstacles that may decrease it. Following paragraphs will present a more in depth discussion for each prominent driver.

### 4.1. Trust

The results have revealed a trend in affirming the positive correlation with supplying quality information and procedural justice, and levels of mutual trust between the community and the developer. The research suggests that a developer should always prioritize establishing mutual trust with the community. Generally, increasing mutual trust is done through community participation and the dissemination of information. To increase the probability of community acceptance, a transparent process builds mutual trust with residents and allows them to stay informed at various stages of development. Conversely, a lack of transparency contributed to rejection and significant opposition by residents to the development of REP in their community. Significant disapproval by the community is linked to a lack of opportunities for community involvement and misinformation about REP. Transparent communication is crucial in establishing trust and can affect community perceptions of renewable energy. For instance, where there is no communication between residents and developers, communities tend to view this strategy as deceptive and this creates distrust. It is crucial in maintaining community acceptance to engage with locals and establish mutual trust through open communication and opportunities for participation as early as possible in the development of REP. The level of communication and participation depends on the individual REP because certain constraints may prevent a transparent process from the early stages of the REP. The involvement of the public interested groups (i.e., local communities that often may oppose the initiative) in the early stage of project development allows the REP developer to consider the specific requests of local communities and develop a mutually beneficial partnership that will facilitate the construction of new REP installations in the area and its future operation. Generally, transparent communication with the public about a developer’s REP plans from the early stage of the project development will provide an opportunity for the local community to participate in the planning decisions and will likely increase social acceptance. Conversely, the lack of collaboration and transparency from developers has always led to a strong opposition from several national and local non-governmental organizations (NGO) causing both an economic loss for the developer and a missed opportunity for the local communities to move their local economy towards an advanced business-oriented green model to foster a more efficient circular economy. An important method of promoting participation and establishing trust is the creation of a local supporters’ network. This network can be made up of prominent local actors who already carry out with them some level of community trust and are publicly supporting the REP. Consequently, this local supporters’ network can disseminate information into the public sphere more organically, associate the REP with trusted and positive actors, and counterbalance any opposition networks that may be formed [12,13,16].

Public involvement in the planning process is crucial for the acceptance of a REP. While substantial public participation can be bothersome for developers, the involvement of the community can be minimal. Creating opportunities for residents to participate in the development of REP in small ways can significantly improve public perceptions. For instance, information sessions, public forums, and listening to public opinions and concerns can greatly improve perceptions of REP, dispel any misconceptions, and concerns that would have arisen inevitably over the planning process can be confronted and mitigated. Participatory democracy constitutes nowadays a key item in the European model of social development. In the Treaty of European Union (TEU), amending the Treaty of Lisbon (13 December 2007), complementarity between representative democracy and participatory democracy is established. Participation becomes a right of citizens and subsidiarity is a cornerstone of participatory democracy. Public participation in planning and development also quite often leads to the dissemination of quality information into the public. Most of the literature analysed confirmed correlation of public participation and a project’s success. Generally, a strong opposition network seems to arise as a result of exempting the local population from either participating in the decision-making process or simply supplying information about the proposed RE.

Public debate is an operating method engineered by a commission of specialists with proven experience and authority, capable to give the necessary guarantee of credibility to the entire discussion, intended to involve citizens and inhabitants in the process of developing major actions regarding the territory: it ensures full and transparent information about an action in the design stage to all stakeholders, offering them the opportunity to express their opinion, both as individuals and as organized groups.

The literature has shown that the dissemination of information and promoting procedural justice will likely build trust between the community and the developer. A lack of trust in the principal actors of an REP and in the process are a significant barrier to the local acceptance of REP. Many concerns are founded upon mistrust and misinformation such as economic, environmental, and human ecological concern. While there can be legitimate concerns, the results affirm that much of the worry is borne from a lack of knowledge.

### 4.2. Distributional Justice

Distributional justice ensures that the benefits and costs of the project are fairly distributed [22]. The literature suggests that distributional justice is an institutional determinant of social acceptance for every community across Europe. While promoting distributional justice through financial participation alone may not have a large impact on local acceptance [22], it is important that the perceived benefits and costs of an REP are equally distributed among all participants in a community. The results also affirm that financial compensation in some form is a powerful incentive for the local acceptance of an REP that may inconvenience the public. The trend shown by the results is that a financial community benefit will increase the public’s support for a RE plant. The perception of benefits can also be instrumental in increasing local acceptance. For instance, the creation of jobs, even if there are only a few, is perceived as a community benefit that will likely promote the acceptance of an REP.

### 4.3. Siting Issues

The bibliographic survey indicates that many issues related to the siting of an REP can be attributed to the physical aspects of the type of renewable energy. Environmental and human ecological concerns can be unique to a specific REP. For instance, a frequent concern of wind farms is the noise pollution caused by the wind turbines. However, it was also shown that some siting issues are related to emotional and psychological factors such as the rejection of the implementation of an REP because of place attachment or the destruction of landscape that will ensue. Some of the local opposition towards the installation of REP in their community is attributed to the NIMBY phenomenon. This is another psychological factor influencing the local acceptance as it displays a certain level of contradiction where they support RE but not its implementation in their community.

Another key point with siting issue is that REP plants can often exist at very small scale, while conventional energy plants generally consist of larger industrial facilities. Most of the examples provided in recent literature [43,44,45] can likely be installed very close to residential locations, thus increasing the NIMBY phenomenon.

### 4.4. Socio-Demographic Factors

From the analysis of the cited papers it appears that many socio-demographic factors can influence multiple aspects of local acceptance. The results have shown that socio-demographic factors affect the local acceptance in varying ways depending on the specific community and the country. For instance, different countries have varying political atmospheres which can deeply impact the level of trust in the process, the willingness to adopt RE, and the tolerance for community costs, which are all determinants of social acceptance. There is no consensus on the direct effect of specific socio-demographic factors as they have varying impacts depending on State, however, it is important to recognize that they do have a certain effect on the local acceptance of REP.

## 5. Limitations of the Study

Comparative research is limited by a number of factors. Firstly, there are differences in the meaning of social acceptance and the method of collecting data sets for every study in various countries. Furthermore, every European country has unique circumstances that may affect the local acceptance of REP in a community and are not considered by each study, such as the political atmosphere. The main drivers of social acceptance are complex and are beyond the Not in My Backyard Phenomenon (NIMBY), an oversimplification of the rejection of RES in communities. This study aims is limited to the most common and frequently mentioned influences in the case studies. This qualitative comparative analysis cannot prove the validity of a generalization on the drivers of social acceptance; however, it can provide persuasive evidence that such drivers exist [46]. Despite the limitations of the study, it produces an insightful generalization on the drivers and barriers of local acceptance across Europe.

## 6. Conclusions and Policy Implications

The EU has made plans to adopt more RE in the coming years. As social acceptance is a significant barrier in the implementation of REP, governments must consider the general trends in local acceptance and create a framework that will increase the probability of local acceptance and reduce the chances of an opposition network that will hinder the development of an REP. Trust in principal actors remains a significant driver in local acceptance. It has been demonstrated that to foster acceptance of renewable energy projects, trust in local authorities and developers must be gained by the public. This trust should be built through a transparent process along the full chain from planning to development and plant’s operation. Additionally, the provisioning of quality information and allowing the public to participate and voice their concerns in the planning process is an institutional factor in the local acceptance of REP. The information shared with the public should be of high technical quality, including data related to the economic and environmental impacts including cost-benefits analysis of technological solutions adopted by developers for a specific REP. Factors such as education and income of residents are key factors affecting public acceptance of any REP installation.

Moreover, the outcome of the cost–benefit analysis was identified as the most influential factor for public acceptance. Economic and social advantages due to the installation of a REP would certainly favour public acceptance and would foster. Compensation measures such as reduced energy costs for residents, the development of recreational infrastructures, increased environmental quality, and foreseen increases of the tourism industry are all factors that have a strong influence on public opinion and acceptance.

## Figures and Tables

**Table 1:** Summary of materials used for the quality comparative analysis.

Study	Nation(s)	Summary/Goals
[22]	Switzerland	A hypothetical wind park project which implements a different financial participation model is presented to four samples of participants to determine which participation model promotes the highest level of local acceptance.
[23]	France, Ireland, Italy, Spain, United Kingdom	The study evaluates the level of citizen engagement for six participatory business models (BPMs) across six European communities using qualitative and quantitative tools to determine the indicators that boost acceptability.
[24]	Austria, Germany, Italy, Switzerland	An acceptance survey, conducted in European communities, with 500 respondents to collect their general opinions on renewable energy and related technologies such as renewable energy communities.
[25]	Italy	Survey with 152 respondents from eight municipalities in rural southern Italy to investigate the key factors influencing the perception of risks and benefits of a biomass combustion plant.
[26]	Portugal	Studies conducted in renewable energy facilities in Portugal to determine community perceptions of energy infrastructures during construction and after its implementation.
[27]	Switzerland, Germany, Austria	Survey among 2104 participants to analyse their opinions on the opportunities and challenges of implementing distributed energy systems.
[28]	Denmark	Large-scale questionnaire survey on public perceptions towards planned local near-shore wind farms in Denmark to provide a socio-cognitive account of concerns and opposition.
[29]	Germany, Italy, Latvia, Norway, Poland, Spain	Qualitative analysis to determine the level of local acceptance of wind energy from municipalities across Europe.
[30]	Regions of Europe (northern, western, central, southern)	A quantitative study on perceptions and factors that influence the social acceptance of wind energy in European regions.
[31]	Germany, Portugal, Sweden	Interviews of 270 participants to determine the importance of 25 qualifiers in public opinion about run-of-the-river hydropower.
[32]	Germany	A questionnaire survey with 1247 respondents to determine the strongest psychological and social factors of local and general acceptance of the nearby implementation of three different energy technologies.
[33]	Slovenia	Guided discussions with 28 participants to determine the public perceptions of solar power plants’ noticeability in landscapes.
[34]	Poland, the Czech Republic	Two questionnaire surveys were conducted in two municipalities with 232 respondents and expert interviews with 19 participating local and regional stakeholders to determine local perceptions of anaerobic digestion (AD) plants.
[35]	Germany	A comparative analysis of existing literature and independent survey of residential preferences, with the goal of exploring the preferences and trends in public acceptance of various RES in Germany.
[36]	Austria	Case study of attitudes toward potential wind expansion sites in Austria to identify reasons and patterns for acceptance and opposition to wind energy.
[14]	Switzerland	Survey of over 500 Swiss citizens living near biogas plants to discover what factors lead to high and low acceptance of renewable energy technology.
[15]	Italy	A specific case study dealing with a proposed biogas plant in a small community in an alpine region of Italy and its subsequent failure to be realized. The aim was to discover the reasons the biogas plant was opposed by the resident population.
[12]	England	A specific case study dealing with the failed implementation of a proposed biomass plant in England, with the goal of discovering what made the public reject this proposal and the steps that could have been taken to resolve these issues.
[13]	France, Germany	Compilation of five case studies concerning the successful implementation of wind parks in various regions of France and Germany to discover what strategies were taken to ensure successful implementation and public acceptance of these wind parks.
[16]	Denmark, England, Wales	Compilation of eighteen case studies of successful implementations of wind energy projects in England, Wales and Denmark. The study identifies the commonalities and trends involving the relationships between participation, network stability, and public acceptance and planning success.
[37]	Germany, Italy, Latvia, Norway, Poland, Spain	A survey carried out in six European countries to determine the community acceptance of wind farms based on a variety of factors.
[38]	Germany	Compilation of many case studies of public acceptance of various renewable energy technologies to analyse the social aspects and determinants of public acceptance of RES.
[39]	Spain	Case study of an ocean wave plant to analyse community acceptance of ocean wave energy and understand public attitudes toward it.
[10]	Finland	Survey concerning the public opinion and knowledge of renewable energy in Finland.
[8]	Portugal	Survey to evaluate public opinion and knowledge of renewable energy sources in Portugal.

**Table 2:** Varying results and opinion of trust in governance and procedural justice on social acceptance.

Study	Consensus
[22]	The study states that acceptance could increase if a community participated in designing financial participation models, however, project leaders may not always be willing to discuss the specifications of financial participation with the local population.
[23]	A qualitative analysis of 44 in-depth interviews across six European communities reveals that participants desire real agency beyond consumer empowerment in the implementation of large-scale RES projects. Respondents expressed the need for clearer information on community energy projects. The study introduces six participatory business models (PBMs) where the citizen participation potential of each PBM on a five-point Likert scale is evaluated. Respondents will then discuss their opinions based on the PBM. Generally, local control and community benefit potential were the most influential in increasing acceptance. Local control is described as opportunities for local citizens to engage in the development of RES. PBMs that scored a high level of local control often received more positive reactions. Overall, the most successful PBMs are those that score highly for all eight indicators; local control, local focus, local ownership, community participation, community benefit potential, infrastructure change potential, wealth-generating potential (local), and wealth-generating potent (extra-local).
[25]	A 14-item questionnaire is conducted with 152 respondents that are consumers, farmers, or professionals wherein each item is given a rating on a five-point Likert scale. The research confirms that a major barrier has been distrust of new technology and that by providing information and communication, the level of mutual trust with a company is increased. In the framework of a small-scale agro-energy system, two determinants influence local acceptance: economic, environmental and social benefits and reassurances from the energy company management. While there is generally high approval for RES projects in communities, there are often concerns that may undermine its implementation such as doubts about perceived benefits and possible risks. Reassurances from energy company management increase trust and mitigate concerns from locals; informing the public of policies that will preserve their quality of life is one of such reassurances. Moreover, the perception of converting agricultural residues into energy for local needs as an “opportunity” for the community increases its acceptance. The general concept of a small-scale agro-energy system is accepted so long as public events are organized and participants are enabled to express opinions and feelings. For instance, the existence of a monitoring committee made up of citizen representatives increases the level of trust in company management.
[26]	The case study focuses on the perceptions of local stakeholders and residents on wind farms and a photovoltaic solar power plant in Portugal. Residents that are against RES mention concerns that are based on lack of knowledge and require assurances of environmental harmlessness. Providing quality information, for example on negative environmental impacts on animal welfare or tied to waste and the fate of disused material the new RES plant may have, helps mitigate concerns based on misconceptions of RES. Information also affects individuals’ perceptions and connotations of RES which influences their level of acceptance. For instance, wind farms and solar power plants are seen as less damaging than fossil fuel or nuclear energy which affirms the perception that RES offers more environmental benefits.
[27]	On a five-point Likert scale, participants had to indicate the correctness of a statement in a questionnaire (1 = “not correct at all”, 5 = “fully correct”). This study reveals that most participants agree that they have too little knowledge about local, distributed energy systems (DESs) which utilize RES, With the average rating for this statement being 3.7, meaning most participants feel that it is correct. This reveals a degree of uncertainty among the respondents. The results demonstrate that the acceptance of DESs is driven by “perceived opportunities” and that in-depth information campaigns aimed at local citizenry may help in increasing RES and DES acceptance.
[29]	This study assesses 30 drivers of social acceptance for 10 wind energy companies across Europe and determines which drivers are most relevant. It was found that transparent communication was the social acceptance driver most represented among all partners. However, active participation, whether direct or indirect, was less common in only 4 out of 10 practices. It is emphasized that active participation is more burdensome because it requires frequent responses and actions by local citizens. Credibility and trustworthiness are fairly represented among the practices appearing respectively in 6 and 5 cases out of 10. Effective formal participation was found in seven cases. This is described as opportunities for locals and stakeholders to engage in a manner prescribed by statutory regulation such as local referenda, public consultations and hearings, public meetings, and public surveys. This participation has a more positive influence on local acceptance if it is genuine.
[30]	With 108 responses from 33 countries, among the most relevant reasons for resistance against wind energy projects is lack of trust, encroachment into the landscape, and environmental concerns. The study includes a quantitative analysis where participants and experts use a five-point Likert scale to indicate which items they objected to the most. Lack of trust is the main reason for resistance in central and eastern Europe and is the second or third most important factor for northern, western, and southern Europe. Much of the literature suggests that resistance against RES is caused by a lack of procedural justice. The study also evaluates which aspects of strategic planning are most important. Communication quality is among the principal determinants of social acceptance.
[31]	Guided discussions with participants have revealed that many were concerned about the efficacy of run-of-the-river hydropower and the technology’s impact. They also demonstrated misconceptions about ecological measures. The study emphasizes the need for policymakers to re-evaluate the way information is disseminated and the benefit from increased monitoring by operators.
[32]	A questionnaire with 1247 respondents signaling their approval or general acceptance on a five-point Likert scale revealed the public perception of hydrogen fuel stations (HFS), biofuel production plants (BPP), and stationary battery storage (SBS). Generally, trust in one’s municipality has a significan positive t effect on local acceptance and little or no effect on general acceptance. This study indicates that the perceived problems of the current energy system have a significant effect on the general acceptance of renewable energy technology (RET). It is suggested to raise awareness of the issues concerning the current energy system and making a comparison with RES and RET to increase general acceptance.
[34]	The methodology consists of quantitative and qualitative analysis through a questionnaire and semi-structured interviews with local stakeholders. The study focuses on biogas as renewable energy and assesses the embeddedness of anaerobic digestion plants (AD) plants. Participants from Poland and the Czech Republic express that support for local institutions and cultural events is crucial in the embeddedness of the AD plant. Embeddedness is defined as a complete immersion into social, cultural, economic, and political life. The results show that a lack of local participation from residents and stakeholders harms the development of AD plants in Central-European countries. Without transparent communication or a participation model, the opinions of locals are not heard and it derails local planning. Furthermore, negative opinions about the RE frequently related to respondents that have never visited an AD plant. The study concludes that knowledge about biogas directly determines the acceptance of a RES.
[36]	Based on 28 semi-structured interviews with experts, stakeholders, wind park developers, environmental and nature conservation groups and others, the involvement of citizens and local stakeholders in planning and siting decisions is considered instrumental in the implementation of RES such as a wind farm. All respondents agreed that project leaders must inform the municipality of the specifications of the project. For instance, the number of turbines, the location, local investments, environmental and health consequences. Informing the public of opportunities for engagement is also critical. This study found that public opinion polls, while increasing local engagement, do not allow fair negotiations or conflict resolution.
[14]	The study found that local acceptance of biogas plants in Switzerland is highly affected by public trust toward the plant operator. Supplying the local population with quality information significantly increases the local acceptance and also enhances trust in the developer, which enacted a positive feedback loop increasing public acceptance even more. However, the level of direct participation of the local community did not affect local acceptance. The author dismisses this finding as not indicative of the general trend since Switzerland already has so much direct public involvement in the government.
[15]	Strong public opposition led to the shut-down of a bioenergy plant and the study asserts that adopting an inclusive and transparent process that would involve all interested parties could increase the social acceptance of a REP. A strong opposition network seemed to rise as a result of exempting the local population from either participating in the decision-making process or simply supplying information about the proposed RE. The information regarding a proposed biogas plant and its costs and benefits was not distributed to the general public until the project gained approval from the local government. While local farmers were involved in the planning of the plant, the majority of the local population was not considered by the private developers and local political institutions.
[12]	The study conducted in England affirms that gaining public trust requires transparency. As the developers were not able to disclose their REP to the public because they were engaged in a highly competitive bidding process, in the results was a lack of trust and consequent lower acceptance.
[13]	This study asserts that local developers are more trusted by local communities compared to non-local developers. There is the notion that local communities and local developers share the same social and economic risks and advantages.
[16]	It was found that projects with high levels of participatory planning are more likely to be both socially accepted and successfully implemented.
[37]	This study determines the most relevant barriers to community acceptance according to stakeholders. Among the important contextual factors of community acceptance are trust in information, trust in key actors, transparency, and trust in process.
[39]	This case study of an ocean wave plant identified trust as a key determinant factor for the successful adoption of wave energy infrastructure. It was observed that trust must “flow in two directions”, between the developer and the local stakeholders for the increased likelihood of the public accepting an REP. The study shows that providing opportunities for locals to participate in the decision-making process can be vital to the success of the REP, and also concludes that adequate information about the REP is the main factor in increasing trust. Active and early consultation with stakeholders, and provisioning of full and detailed information to the community aimed at creating meaningful social involvement, is crucial for the successful implementation of REP.

**Table 3:** Comparing the results of the effect of distributional justice and economic interest on social acceptance.

Study	Consensus
[22]	Distributional justice is the main institutional factor for community acceptance. It was hypothesized that a financial participation model could increase the acceptability of a wind park because it promotes equity in the distribution of the project’s benefits and costs among residents. The study implemented three different financial participation models on a hypothetical wind park and determined on a five-point Likert scale the Swiss residents’ level of acceptance. This included an investment model based on voluntary share purchasing, another model based on bond purchases, and the last model was a collective plan with a wind resource tax and compensation payments. It was shown that while a financial participation model of collective nature and low-risk aversion was preferred, there was no significant increase or decrease in local acceptance between the wind park without a financial participation model and those that were given treatment. There is only some experimental evidence to demonstrate that promoting distributional justice through financial participation models, such as a wind resource tax, increases acceptability, and even then not substantially. The consensus is that financial participation alone is not an influential factor in social acceptance, however, it should still be considered to positively influence investors with high-risk aversion and other target groups. For instance, risk-averse people are significantly more likely to accept a wind park when a wind resource tax exists. Moreover, centre-progressive voters are more likely to accept when any financial participation model is implemented. The study concludes that distributional justice remains an institutional factor in local acceptance and the perception of equity in benefits and costs among locals is crucial.
[23]	This study revealed that community-oriented projects would receive greater local acceptance with a substantial community benefit beyond support for local events. The acceptability would increase if individuals in the community could benefit financially, even in a small part.
[24]	Interestingly, this study claims the results support the notion that local consumers are willing to pay higher electricity rates in a renewable energy community configuration that includes a substantial proportion of solar-generated power.
[25]	Economic, environmental and social benefits for the community are among the most important determinants of social acceptance in a small-scale agro-energy system. A large influence in local acceptance is citizens’ expectations of a reduction of taxes and energy costs since local energy demands are satisfied. Another perceived benefit that increases social acceptance is the potential contribution to the economic development of the community and attaining energy self-sufficiency.
[26]	The socioeconomic impacts of RES greatly influence the level of acceptance in a community. This study revealed that a financial benefit, such as 2.5% of annual revenue awarded to municipalities, offers a strong incentive for local authorities to accept wind farms or other RES. The creation of employment is another economic benefit that is greatly valued by locals, primarily by stakeholders.
[29]	Contrary to the first case study, financial participation has been a common driver for community acceptance, as shown in 8 cases out of 10. The results show that a positive effect on the local economy is considered “important” or “highly important” for all practices. For instance, the creation of jobs in the local economy is a driver of acceptance.
[30]	Lack of national incentive is largely the reason for rejection in three regions of Europe: northern, central and eastern, and southern.
[31]	The implementation of run-of-the-river hydropower faces social acceptance barriers. Most participants support hydropower because of its environmental benefits and its contribution to achieving energy self-sufficiency. However, respondents also agree that locals should receive part of the profit or receive financial compensation directly or indirectly through economic development. For instance, they value low energy prices and job creation in the region.
[36]	This study reveals that the distribution of costs and benefits among the local community can be a controversial issue. In this context, operators of wind farms must compensate for the negative effects or economic losses to the municipality. However, some respondents expressed distrust and claimed that payments are used to buy people’s votes or interests. Most respondents agreed there was a need for transparency regarding the distribution of costs and benefits. For instance, revenues should be set aside for disclosed specific purposes. Experts have indicated that parties should also compensate neighbouring communities that are affected by the wind farm.
[13]	The perceived benefits and costs of an REP to the community is a crucial determinant of local acceptance. Using the cost-benefit analysis to determine the negative potential effects on the local economic sectors that a wind farm would cause, residents considered the benefits of the wind parks against the potential negative cost to their industry.
[37]	The study affirms that the following determinants have a small but positive impact on acceptance: impacts on individuals’ economic situation, and impacts on local profits and income generation.
[38]	The costs and benefits for the local community associated with a REP is certainly the most critical factor in building public acceptance. Economic evaluations performed by local communities and consideration of local needs associated to a REP are the most influential factors on the public acceptance of a REP installation. Depending on whether the project would lead to economic gains or losses, the public would either accept or oppose the project development.
[10]	A strong majority (62%) of the respondents were willing to pay higher costs to obtain green energy. Additionally, nearly 30% of respondents were willing to pay up to 10% more in taxes to obtain green energy. These results prove that some raw economic factors, at least the price of electricity, are not the only factors that affect public acceptance, and that some populations are willing to sacrifice their economic interests for environmental interests.
[8]	The study reveals that negative economic impact of a RE technology can “strongly reduce its acceptance.” For instance, the national attitude towards hydropower projects was more negative when the perceived costs of the technology are higher. However, losses and benefits of a REP are not only financial. The costs and benefits of a REP can be measured in a variety of forms such as the visual impact on the landscape, the economic impact on tourism, decreased values of surrounding properties, the creation of a number of jobs, an increased or lower price of energy, an equal distribution of the benefits or costs among the community, siting of a project on public or private land, environmental impacts on surrounding areas, and health consequences.

**Table 4:** Comparing the results of the effect of siting and physical attributes of renewable energy (RE) on social acceptance.

Study	Consensus
[24]	The study found that a renewable energy community configuration that includes gas plants significantly decreases social acceptance and increases the likelihood that respondents prefer the status quo. There is a substantial negative effect of overhead power lines due to its assumed detrimental health effects. The results have shown that avoiding additional electricity transmission through visible overhead lines is important in maintaining levels of local acceptance. This study reveals that considering the effects on human health is an important aspect of siting RES projects.
[26]	In a case study on wind farms and solar power plants in Portugal with 150 participants, the negative effects on landscapes are frequently mentioned during the interviews. RES in localities is criticized by locals for the negative landscape transformation which is often due to “place attachment” or a negative perception of the energy landscape relationship. The concept of “place attachment” is defined as an emotional or cultural attachment to a landscape and its spatial elements. However, the research also indicates that stakeholders are less concerned about landscape or siting issues such as the aesthetic or disturbance to spatial elements.
[28]	The research provided described a renewable energy siting controversy on the differing attitudes to RET in principle and local practices. A survey targeting residents facing near-shore wind farms provides qualitative analysis and demonstrates the theory of cognitive polyphagia. Participants demonstrate a degree of internal contradiction with their statements. For instance, the notion of being positive about wind farms but wishing to relocate them describes an uncomfortable level of dissonance. This statement also affirms the energy siting controversy that there may be more support for RE siting in already-developed areas. The study indicates that emotional responses are an important consideration in the context of RES siting.
[29]	The impact on the landscape is not a common consideration for wind energy companies across Europe, appearing in only 4 of 10 practices. This is contrary to other studies that demonstrate that the negative impact on a landscape largely influences the public’s perceptions and approval of RES. This study notes that the main concern with wind turbines is its acoustic emissions. The impact on biodiversity and life is considered by four practices as drivers of social acceptance. It remains that environmental concerns and the impact on the landscape are important aspects of siting RES.
[31]	Residents’ willingness to live near a RES sometimes depends on the individual circumstances of a specific RE. For instance, this study reveals that respondents do not mind living close to a hydropower plant so long as it protects natural ecosystems and citizens’ well-being. Ensuring the hydropower plant is not negatively affecting the free flow of rivers and implementing a framework for flood protection are steps taken to preserve biodiversity and the community’s quality of life.
[33]	This qualitative analysis on the public’s perception of solar power plants (SPP) indicates that SPPs are a very noticeable element in the landscape and 42% of 28 participants perceive a negative connotation with SPPs whereas only 27% describe a positive connotation. Most of the participants’ comments are negative and describe the destruction of landscape character. They perceive an unpleasant contrast from a highly artificial and technical object in an agrarian landscape.
[36]	The qualitative analysis indicates that the impact on the landscape scenery is highly influential in local acceptance. The study affirms the notion that locals perceive the destruction of landscape scenery by wind turbines. Wind farm operators argue that the younger demographic is increasingly accustomed to an “artificial” or more technological environment and would not perceive wind turbines as a negative impact on the landscape. An important concern among experts and stakeholders are the negative impacts on wildlife conservation and nature. These respondents were also concerned about the associated expenses and planning risks. Participants also expressed concern for the impacts on human ecology. In the context of wind farms, those impacts are the noise, large shadows, and ice shedding. The study reveals that impacts on human ecology considerably influences local acceptance because they can hardly be resolved by expert knowledge or deliberation.
[14]	Some issues related to siting of a RE are caused by the physical attributes of the REP. In this study, an unpleasant smell from the biogas plant was one of the most frequently cited complaint from residents living near biogas plants.
[12]	This study confirms that siting issues related to biogas plants pertain to its physical attributes. The disrupting smell was a common complaint amongst residents with the added concern that it would decrease property values in the areas surrounding a biogas plant. These are concerns that lower the local acceptance of biogas plants.
[13]	The visual impact of a wind farm was one of the major concerns among residents in 3 out of 5 cases included in this study. In one instance, the community’s concern was primarily related to the negative impact on the wine and tourism sectors which represent an important source of income in the region. This demonstrates the impact of the physical characteristics of wind farms on local acceptance when sited in a community.
[37]	Factors related to environmental impacts such as the impact of an REP on the physical environment, biodiversity, and wildlife have a clear negative impact on community acceptance.

**Table 5:** Comparing the results of the effect of socio-demographic factors on social acceptance.

Study	Consensus
[22]	The study concludes that political attitude highly influences local acceptance of RES. For instance, conservative and centre-progressive voters tend to reject and oppose initiatives that favour the development of RE like wind energy. The data shows that political attitude has a significant, medium-size effect on acceptance in general, however, acceptance is not dependent on political attitude. One exception is that centre-progressive voters were more likely to accept wind energy when a financial participation model was implemented like the wind resource tax.
[24]	This study determines the marginal effect of various factors on the respondent’s willingness to transition to a renewable energy community and adopt various RES. The study indicates that respondents in the age category 35–65 have a higher propensity (1.5%) to choose the status quo, compared to individuals in the 20–35 age group. However, those over 65 years of age do not seem to have the same effect. It was also noted that households with kids are less likely to prefer the status quo and are more willing to transition to a renewable energy community. It is hypothesized that these individuals are perhaps planning for the longer term. Additionally, the political support of a local government has a positive effect on local acceptance. However, increased national and EU level political support has no effect on local acceptance.
[35]	This study found that age and education were the most relevant socio-demographic variables to acceptance of RE technologies. One of their findings highlighted that individuals belonging to an older age group or that were less educated had a significantly lower willingness to adapt their lifestyle and pay an increased price to reduce their ecological footprint.
[8]	This found that residents had generally positive attitudes towards REP, yet also found that higher education levels correlated positively with increased awareness about renewable energy technologies, and that lower education and older age is correlated with higher opposition to various REP.

**Table 6:** Summary of the discussion on prominent drivers.

Prominent Driver	Sub-Category	Notes
Trust	Information exchange	Developers should share transparent and comprehensive informations
	Public involvement	Opportunities should be created for residents to be involved in the development process
	Procedural justice	Fairness should be guaranteed in resolving disputes
Distributional justice	Fair distribution	Costs and benefits should be fairly distributed between residents and developers
	Compensation	Direct or indirect financial compensation may be a good incentive
Siting issues	Physical characteristics	Residents may have issues with potential environmental or health impacts depending on the physical characteristics of the REP
	Emotional factors	Attachment to specific places may be a factor
Socio-demographic factors	Political atmosphere or community characteristics	Effects vary by country and are still not easy to predict
